# Pediatric Chronic Tracheostomy Care: An Evaluation of an Innovative Competency-Based Education Program for Community Health Care Providers

**DOI:** 10.3389/fped.2022.885405

**Published:** 2022-05-31

**Authors:** Jenny Y. Shi, Julia Orkin, Catharine M. Walsh, Stephanie Chu, Krista Keilty, Sandra McKay, Cora Mocanu, Adam Qazi, Munazzah Ambreen, Reshma Amin

**Affiliations:** ^1^Paediatric Respiratory Medicine, Hospital for Sick Children, University of Toronto, Toronto, ON, Canada; ^2^Complex Care Program, Hospital for Sick Children, University of Toronto, Toronto, ON, Canada; ^3^Division of Gastroenterology, Hepatology and Nutrition, Department of Paediatrics and the SickKids Research and Learning Institutes, Hospital for Sick Children, University of Toronto, Toronto, ON, Canada; ^4^Connected Care, Hospital for Sick Children, University of Toronto, Toronto, ON, Canada; ^5^VHA Home HealthCare, Toronto, ON, Canada

**Keywords:** home nursing, tracheostomy care, education program, pediatrics, simulation education in nursing

## Abstract

**Objective:**

To evaluate the immediate and sustained knowledge retention and sense of self-efficacy of homecare nurses following completion of a standardized competency-based tracheostomy education course. Safe discharge of children requiring tracheostomy with or without ventilation relies on the competence of homecare nurses.

**Study Design:**

Pragmatic, randomized controlled trial of 44 homecare nurses. Participants were randomized into the intervention group (*n* = 21), which received the tracheostomy course, or the control group (*n* = 23), which received an enterostomy and vascular access course. Multiple-choice question (MCQ) knowledge assessments and self-efficacy questionnaires were administered to both groups pre-course and post-course at 6 week, 3 month, 6 month, and 12 month follow-ups.

**Results:**

Twenty participants in the intervention group and 19 in the control group were included. Four withdrew from the study and two crossed over from the control into the intervention arm. The change in mean self-efficacy scores (total score = 100) was significantly higher in the intervention group than in the control group at 6 weeks (intervention (mean ± SD): 18.6 ± 14.5; control: 6.6 ± 20.4; *p* = 0.04) and 3 months (intervention: 19.6 ± 14.2; control: 5.2 ± 17.0; *p* = 0.007), and trended higher at 6 months (intervention: 18.0 ± 14.5; control: 6.9 ± 24.1; *p* = 0.1). The change in mean MCQ assessment scores (total score = 20) trended higher in the intervention group than in the control group at 6 weeks (intervention (mean ± SD): 1.8 ± 2.2; control: 1.6, ± 2.9; *p* = 0.8).

**Conclusions:**

Homecare nurses who attended the tracheostomy course demonstrated a higher sense of self-efficacy at long-term follow-up.

**Clinical Trial Registration:**

www.ClinicalTrials.gov, identifier: NCT04559932.

## Introduction

Advancements in health care and technology have contributed to the growing prevalence of children requiring chronic tracheostomy tubes (1). These patients make up a part of the growing population of children with medical complexity (CMC), defined as patients with characteristic patterns of substantial family-identified needs, chronic conditions associated with medical fragility, severe functional limitations often associated with technology dependence, and high health care use (2). As a result, these children are at significant risk of morbidity and mortality and generally have a high burden of healthcare utilization and spending across their care continuum (3).

Safe discharge of children with chronic tracheostomy tubes with or without ventilation relies on the careful coordination and investment of families, homecare nurses, other caregivers, equipment, financial resources, and an interdisciplinary health care team (4, 5). Guidelines from the Canadian and American Thoracic Societies highlight caregiver training and determination of competence as important components in ensuring the safe discharge of children with chronic tracheostomy tubes (5, 6). Improved caregiver competence in emergency tracheostomy management may prevent up to 8–19% of tracheostomy-related deaths in this population (7).

Significant gaps in the knowledge, skills and behaviors required to care for these children have been documented among community homecare nurses (8). Not only do these deficiencies jeopardize the child's safety, but they can also negatively affect communication and trust between homecare nurses and families (9).

Currently in Canada, nursing education curricula includes limited training in pediatric nursing and may not include specific training in homecare or the care of children with medical complexity. Training of homecare nurses is instead the responsibility of the individual nursing agencies and is not standardized. Furthermore, the number of homecare nurses who have expertise in caring for children with tracheostomy with or without ventilation is limited.

To address this gap in expertise and training, our interprofessional group at SickKids in Toronto, Canada, collaboratively designed and implemented a standardized competency-based tracheostomy education course for homecare nurses. While our group has rigorously developed the curriculum and evaluation measures, the course outcomes remained unknown. Our study objective was to evaluate the immediate and sustained knowledge retention as well as sense of self-efficacy of homecare nurses following completion of the course.

## Methods

### Participants

Homecare nurses were either Registered Nurses (RN) or Registered Practical Nurses (RPN) who were recruited from a leading homecare provider organization for children with medical complexity, the VHA Home Healthcare Agency in Toronto, Canada. Eligible participants were new registrants for the competency-based tracheostomy education course who had not previously cared for a patient with a tracheostomy. Participants were also English-speaking. Courses were voluntary, provided free of charge to participants and their time was financially compensated at the same rate as a usual nursing shift. Study participation was voluntary.

### Study Design

We conducted a single-center, 12-month pilot randomized controlled trial at the Hospital for Sick Children, Toronto, Ontario Canada between May 1, 2019 to September 30, 2019. The trial results were reported in accordance with the Consolidated Standards of Reporting Trials (CONSORT) recommendations (10).

### Study Intervention and Control Group

#### The Study Intervention: The Tracheostomy Education Course

The tracheostomy course was developed with collaboration from the Division of Respiratory Medicine, Complex Care, Respiratory Therapy, Nursing Education, Family Advisory Network and SickKids Research Institute in 2016. The curriculum is based on the “KidsVent” checklist of knowledge and skills considered essential for caregiver's competency in caring for children with tracheostomy tubes at home with or without ventilation (11) (Appendix 1). The KidsVent checklist was developed by Amin et al. using rigorous consensus building methods including a Delphi voting process involving 95 Canadian clinical experts to identify domains and items for a knowledge and skills checklist for caregivers of children requiring tracheostomies with or without ventilation (11). The delivery of the course was based on principles of adult learning and includes interactive, small-group teaching, hands-on simulation-based learning stations with relevant equipment, and formal assessments of knowledge and practical skills using written and simulation testing (12). Pre-learning packages were also sent to course attendees at the time of registration to facilitate higher level discussions and engagement on the day of the course (13).

#### The Control Arm: The Enterostomy and Vascular Access Training Course

In lieu of the tracheostomy course, an enterostomy and vascular access training course was offered to the control group to minimize the potential confounder of generalized improvements in self-efficacy that may occur as a result of participating in an 8 h learning opportunity at a tertiary academic institution.

#### Randomization and Allocation of Interventions

One to one randomization and intervention allocation was performed by the nursing agency using a random number generator.

### Study Procedures

Prior to the start of the course, baseline demographic characteristics were collected and a multiple-choice question (MCQ) pre-test (Appendix 2) and self-efficacy questionnaire were completed (Appendix 3).

Thereafter, the MCQ knowledge and self-efficacy assessments were repeated at the 6 week, 3 month, 6 month, and 12 month time points following course completion. The instruments were administered online using Research Electronic Data Capture (REDCap) version 10.2.3, a secure web application for building and managing online surveys (14).

### Study Measures

Immediate and sustained knowledge retention of the homecare nurses were assessed using a MCQ knowledge test that was developed by clinical experts. The test was comprised of 20 questions (maximum score = 20) that included the following major themes required to safely care for a child with tracheostomy ventilation: clinical assessment; safety; general care; troubleshooting; and emergency management.

Self-efficacy, a person's belief in their ability to perform specific tasks, was evaluated using a self-report measure that was developed based on Bandura's self-efficacy theory, consisting of 16 items assessing strength of perceived self-efficacy. Caregivers were asked to rate the strength of belief in their personal capability on a 100-point scale, ranging in 10-unit intervals from 0 (“Cannot do at all”); through to intermediate degrees of assurance, 50 (“Can do it moderately”); to complete assurance, 100 (“Highly certain can do”) (15). The major themes in the survey reflected those in the MCQ knowledge test.

### Study Outcomes

Our primary study outcome was the immediate knowledge retention (MCQ assessment) and change in self-efficacy in the immediate time frame of 6 weeks post-intervention. Secondary study outcomes included mean sustained knowledge retention and change in self-efficacy scores at 3-month, 6-month, and 12-month time points. Additionally, the questions that were answered incorrectly by more than half of the participants were reviewed for prominent themes.

### Statistical Analysis

Descriptive statistics were used to summarize the baseline characteristics of the study participants. Paired and unpaired sample t-tests were used to calculate the change in MCQ knowledge scores and mean self-efficacy sfcores pre- and post-tracheostomy course at 6 week, 3 month, 6 month, and 12 month time points within and between groups. P-values of <0.05 were considered statistically significant. Analyses were performed using Statistical Product and Service Solutions (SPSS, Version 24) and GraphPad Prism 8 statistical software.

A priori sample size calculation indicated that 16 participants in each group was sufficient to detect a clinically important difference of 5 points (25% improvement) on the MCQ knowledge test, assuming a standard deviation of 5 points, using a two-tailed t-test of the difference between means, a power of 80%, and a significance level of 5%. A normal distribution of MCQ test scores was assumed. This number was increased to 20 participants per group (total 40), to allow for a predicted drop-out rate of 20% at 6 weeks. This estimate was based on a similar study by Dorton et al., where healthcare workers completed a same-day 15-question MCQ assessment before and after attending an educational tracheostomy course using patient simulation (16). There was a 35% improvement in scores immediately after the course, but a more conservative estimate of 25% was chosen since the primary outcome measure was at a later time point of 6 weeks (16).

### Ethical Considerations

This study was approved by the institutional review board and the SickKids Research Ethics Board in Toronto, Canada (REB 1000057879). The study was registered with the ClinicalTrials.gov (NCT04559932).

## Results

### Baseline Characteristics

**Figure 1:**
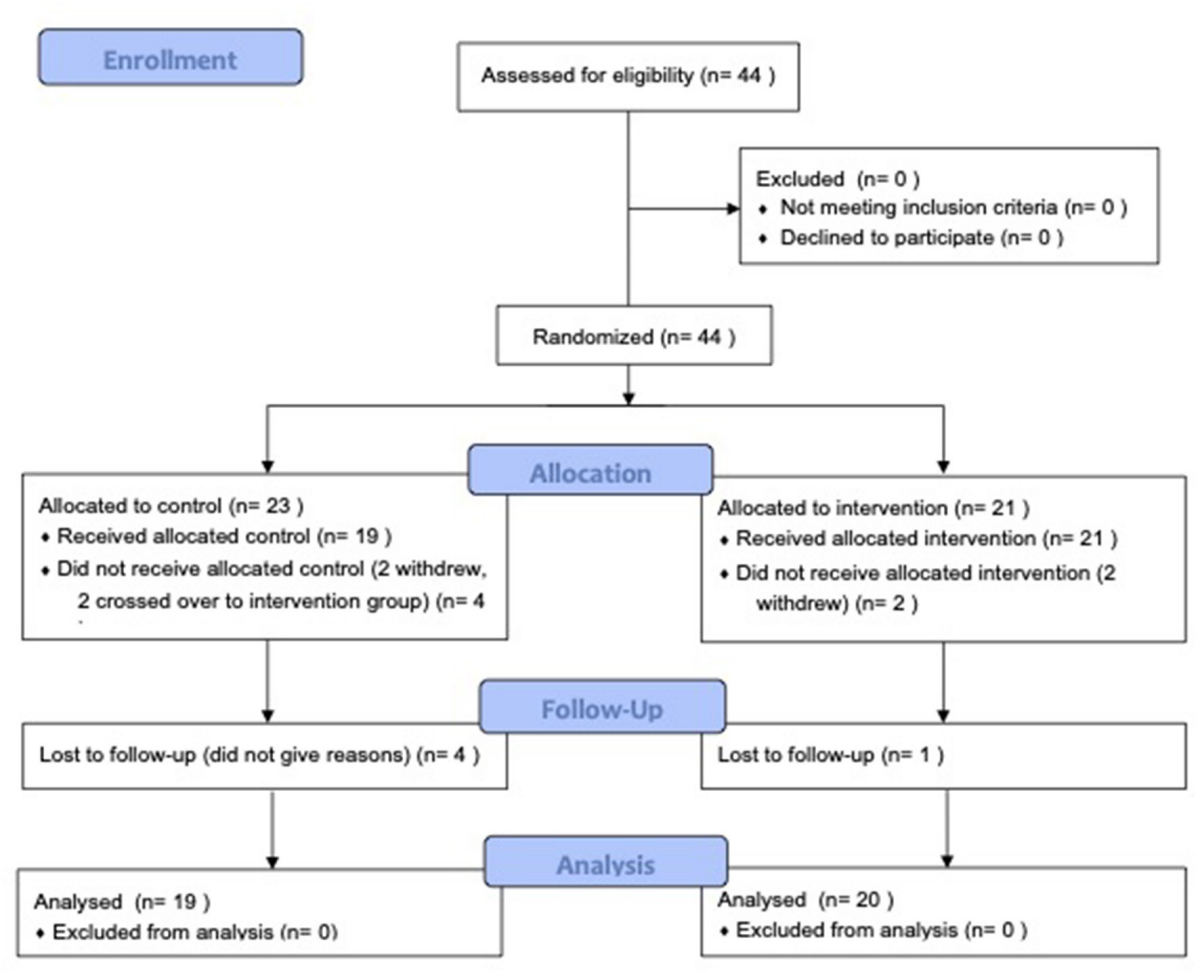
CONSORT flow diagram.

Forty-four participants were enrolled in the study. Twenty-one participants were randomized to the intervention group and 23 to the control group. Two participants who were originally in the control group crossed over into the intervention group at the 3-month and 12-month time points, respectively, at the request of their homecare agency because of the immediate need to provide care for children with tracheostomy tubes in the community. These two participants then re-entered the study starting in the intervention arm and were analyzed as part of this group only. Additionally, one participant from the intervention arm dropped out after the 3-month assessment, while 4 participants from the control group dropped out (one after the pre-assessment, two after 3months, and one after 6 months). Reasons for withdrawal from the study were not provided. Participants were included in the final analysis if they were able to complete at least the 6-week post-course assessments. (See Figure 1: CONSORT Flow Diagram).

There were no differences in the baseline characteristics of the intervention and control group (see Table 1).

**Table 1:** Baseline characteristics.

**Characteristics**	**Control** **(*n* = 19)**	**Intervention** **(*n* = 20)**	**p-value**
Discipline •Registered Nurse (RN) •Registered Practical Nurse (RPN)	7 (37%) 12 (63%)	10 (50%) 10 (50%)	0.41 0.41
Years of experience	2.4 ± 1.4	2.1 ±1.2	0.40
Previous certification^*^	10 (53%)	8 (40%)	0.49
Caring for child with tracheostomy ± ventilation within last 6 months	5 (26%)	8 (40%)	0.36
Prior simulation experience	12 (63%)	12 (60%)	0.84
Baseline MCQ knowledge	11.7 ± 2.6	13.1 ± 1.6	0.05
Baseline self-efficacy score	64.4 ± 23.3	65.8 ± 18.2	0.83

*Baseline characteristics of study participants included in the final analysis. Values are mean ± SD or n (%).*

* *Certification including pediatric, tracheostomy, ventilation, management certification, Pediatric Advanced Life Support, Neonatal Resuscitation Program, Advanced Cardiovascular Life Support, ostomy training, phlebotomy training, unspecified certificates*.

### Multiple Choice Question Knowledge Assessment

Nineteen participants in the control group and the 20 in the intervention group completed the 20-item MCQ knowledge assessment at our primary analysis time point of 6 weeks post-intervention. There was a significant improvement in the mean (± standardized deviation, SD) score of the intervention group from 13.1 ± 1.6 at baseline to 15.3 ± 1.7 at 6 weeks (*p* = 0.002). The mean score of the control group at baseline, however, also improved significantly from 11.7 ± 2.6 to 13.3 ± 1.5 at 6 weeks (*p* = 0.03). The change in the mean scores from baseline to 6 weeks in the intervention group trended higher than the change seen in the control group, though it was not statistically significant (intervention mean 1.8 ± 2.2; control mean 1.6 ± 2.9; *p* = 0.8).

**Figure 2:**
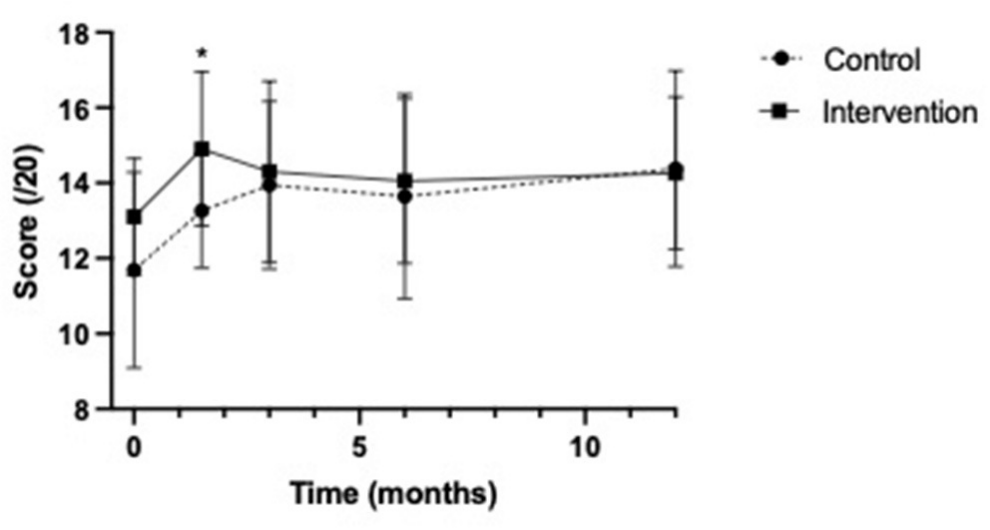
Multiple choice question knowledge test score means. *p-value <0.05, referring to the significant difference in scores between the control and intervention groups at 6 weeks post education.

**Figure 3:**
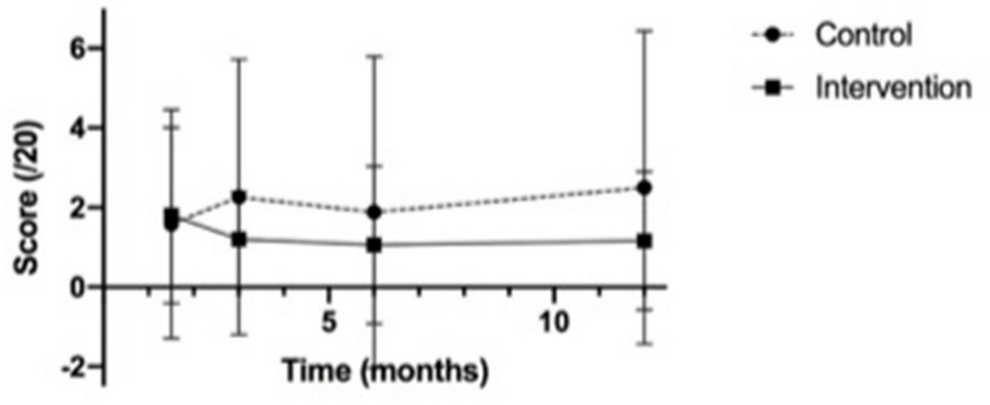
Change in mean multiple choice question knowledge test scores from baseline.

In our secondary analysis at 3 months and 6 months, the mean scores in the intervention group continued to trend higher than those in the control group at all time points (see Figure 2). The changes in scores from baseline were not significantly different between the two groups at any time point (see Figure 3).

We also analyzed which questions were answered incorrectly by more than half of the examinees. The questions that participants answered incorrectly the most in both the intervention and control groups prior to taking the course contained the following themes: indication for tracheostomy, tracheostomy tube maintenance, suctioning, stoma care, equipment troubleshooting, and speaking valve knowledge. Six weeks after taking the course, the same knowledge deficits persisted in the control group, but the intervention group had improved performance on equipment troubleshooting and knowledge about speaking valves. Across groups, the question that was persistently answered incorrectly by more than half of the participants at all time points was “what is the currently recommended frequency of cleaning the stoma?”

### Self-Efficacy Assessment

**Figure 4:**
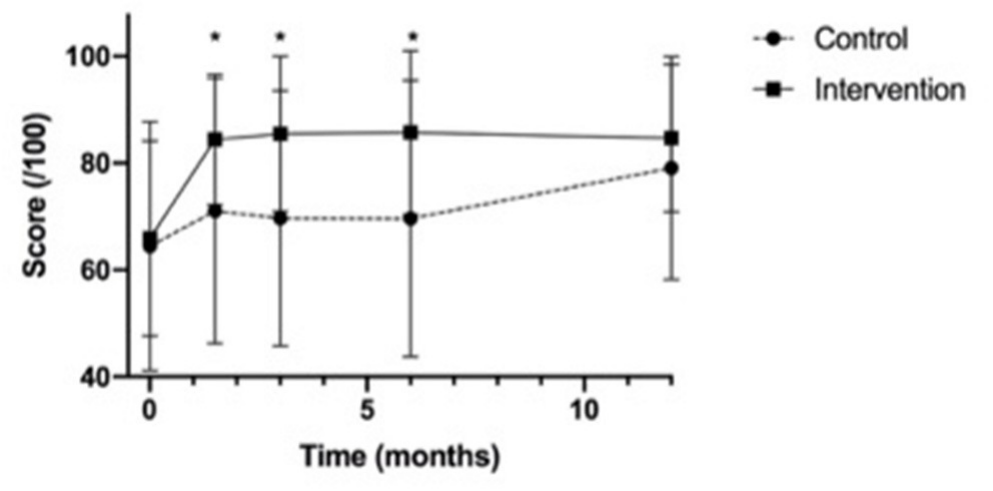
Self-Efficacy assessment score means. *p-value <0.05, referring to the significant difference in scores between the control and intervention groups at 6 weeks, 3 months and 6 months post-education.

Seventeen participants in both the control group (89%) and intervention group (85%) completed the self-efficacy assessments until 12 months post-tracheostomy course. In the control group, one participant was lost to follow-up after the pre-course assessment, two were lost to follow-up after the 3-month evaluation, and one was lost to follow-up after the 6-month evaluation. Overall, the scores in the intervention group trended higher than those in the control group at all time points (see Figure 4: Self-efficacy score means).

**Figure 5:**
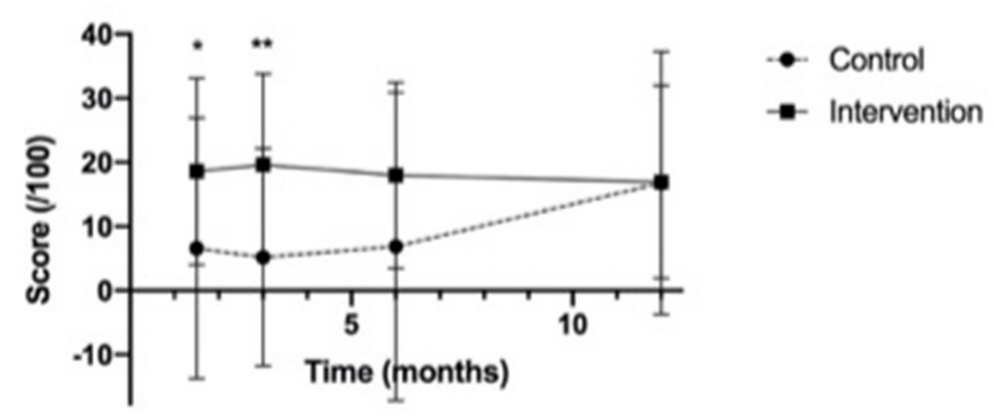
Change in mean self-efficacy assessment scores from baseline. *p-value <0.05, **p-value <0.01, referring to the difference in scores between the control and intervention groups at 6 weeks and 3 months post-education.

The change in mean self-efficacy scores (maximum score 100) were significantly higher in the intervention group than in the control group at 6 weeks (intervention (mean ± SD): 18.6 ± 14.5; control: 6.6 ± 20.4; *p* = 0.04) and 3 months (intervention 19.6±14.2; control: 5.2±17.0; *p* = 0.007), and trended higher at 6 months (intervention 18.0 ± 14.5; control 6.9 ± 24.1; *p* = 0.1) (see Figure 5).

We also qualitatively analyzed the aspects of tracheostomy care at which homecare nurses felt the least confident. The questions that were rated with an average score of approximately 50/100 (“Can do moderately”) and below in both groups at baseline contained the following themes: administering medication via a tracheostomy tube, managing a blocked tracheostomy tube, managing an accidental decannulation of a tracheostomy tube, providing manual ventilation via a tracheostomy tube, and providing bag-mask ventilation to a pediatric client. Six weeks after taking the course, the same knowledge deficits persisted in the control group, but the intervention group did not rate any domain under a mean score of 72/100.

## Discussion

To our knowledge, our study is the first randomized control trial to evaluate the impact of a competency-based tracheostomy training course on knowledge retention and self-efficacy in pediatric homecare nurses in both the short and longer term. The tracheostomy course resulted in a significant improvement in self-efficacy. Importantly, participants in the intervention group felt more confident in their knowledge of tracheostomy care and their ability to troubleshoot emergency and urgent situations involving a tracheostomy tube. Previous studies have found that self-efficacy mediates the relationship between cognitive ability and resulting performance (17). This higher sense of self-efficacy was sustained at 6 weeks, 3 months, and 6 months after training; however, self-efficacy was not statistically higher in the intervention group at 12 months.

The MCQ knowledge assessment scores were statistically higher in the intervention group at 6 weeks post-intervention, but there were no significant differences in the change in scores from baseline. Knowledge in the intervention group particularly improved in questions about tracheostomy equipment troubleshooting and speaking valve knowledge. Knowledge on stoma management remained low, and future course iterations may benefit from the inclusion of more education on stoma care. Although we expected to see a larger overall improvement in MCQ scores, this may be partially due to the format of the assessment. A limitation of the study was that participants were assessed exclusively using a knowledge-based test; however, the course included both didactic and hands-on simulation-based instruction. Future studies would benefit from inclusions of a hands-on, simulation-based assessment of participants' abilities to apply the learned skills. Furthermore, as there was a slight decline in the MCQ scores at later timepoints which may indicate that a refresher intervention or knowledge ‘boost' is needed between the 3- and 12-month time points to maintain the benefits of the course.

Our findings are consistent with existing, non-randomized studies that have assessed the efficacy of tracheostomy educational programs for healthcare workers. Agarwal et al. evaluated the knowledge and confidence level of healthcare providers (pediatric and internal medicine-pediatric residents, pediatric hospitalists, advanced practice RN's) at a tertiary care children's hospital before and after a simulation-based tracheostomy educational program (18). Comfort and confidence levels (5-point Likert scale) improved significantly after completing the course, from median scores of 1 to 4 in different domains of care (performing routine tracheostomy tube care, routine tracheostomy tube change, and emergency tracheostomy tube change).

Similarly, Dorton et al. assessed the self-assessed comfort levels, objective multiple-choice knowledge test scores, and observations of healthcare providers (anesthesia and emergency medicine residents, pulmonary critical care fellows, nurse practitioners, nurses, medical students) in a tertiary care hospital before and after attending a simulation-based educational course (16). Mean ratings of comfort (5-point Likert scale) improved from 3.3 to 4.4 after the training, and mean MCQ scores improved from 56% to 91%. Observational data revealed deficiencies in familiarity with different tracheotomy tubes, speaking valves, and delayed recognition and management of plugged or dislodged tracheotomy tubes. Our study shows that a similar knowledge gap existed in the outpatient setting and suggests that interprofessional providers of diverse backgrounds may benefit from a tracheostomy training course.

Another study by Kun et al. used exploratory online surveys to evaluate pediatric homecare nurses' knowledge of home mechanical ventilation emergency scenarios (8). On average, nurses scored 4.87 (total 10) points and there were no significant differences between nurses with <4 years of experience compared with those with more experience. Ninety-seven percent of nurses from a variety of settings (online training, agency-based training, hospital-based workshops) expressed an interested in more training in this area. This highlights the need for developing enhanced and diverse training opportunities for homecare nurses caring for patients requiring home mechanical ventilation.

Our study did have some potential limitations. First, we had two participants that crossed over from the control group to the intervention because they needed tracheostomy training to care for children in the community. This was unfortunately inherent to the pragmatic nature of our trial. Secondly, all the study participants were recruited from one service provider organization and may not be generalizable across organizations. However, this nursing agency provides care to a significant proportion of children with tracheostomy tubes followed by our institution. Thirdly, five individuals dropped out of the study for reasons not provided. However, our study was adequately powered to test our primary objective despite the dropouts. Another possible limitation was that all the nurses were aware that they would eventually receive tracheostomy training through this study. Consequently, those in the control arm may have been motivated to learn about tracheostomy care on their own while they were enrolled in the enterostomy and vascular access course. This potential “self-training” in tracheostomy care may explain why the control group improved after 3 months despite not having completed the tracheostomy course. There was unlikely to be cross-talk between participants in the control and intervention groups due to the independent nature of their shift work. Lastly, although we were able to ensure that study participants were not assigned care of tracheostomy patients prior to taking the tracheostomy course, we were not able to control for the level of exposure they had with these patients after course completion. As a result, varying levels of real world “on the job” learning may have influenced the results at follow-up.

Further research is needed to evaluate the effect of our tracheostomy course on provider performance to help us better understand both the immediate and long-term impacts on knowledge and skills retention in practice. Evaluation of homecare nurses in their patients' homes, potentially using virtual interfaces, and collection of patient and family experience data would enable assessment of the impact of our course on “real-world” performance. We additionally hope to extend participation to other care providers to assess the impact of the tracheostomy course on family members' and unregulated caregivers' ability to care for children with tracheostomy tubes at home. Furthermore, future studies should assess the broader impact of the tracheostomy course, including workforce retention, career satisfaction and healthcare outcomes, such as recurrent respiratory infections and healthcare utilization.

This study also signals the need to address a gap in nursing education. It suggests that nurses who assume role in homecare lack the foundational knowledge and skills to feel confident and competent in the area of children with medical complexity. We encourage that curriculum for undergraduate and college program in nursing education strengthen the students' learning through simulation and hands-on experience to the care of this high-risk and growing population of children who depend on the routine uses of medical technology in homecare.

## Conclusion

Our study demonstrates the value of a standardized competency-based tracheostomy training course in strengthening the sense of self-efficacy and knowledge in homecare nurses caring for children dependent on chronic tracheostomy tubes with or without ventilation. Enhancing competency in care of this population is important to potentially mitigate significant morbidity and mortality, while fostering safe, high-quality homecare for these medically complex children.

## Data Availability Statement

The raw data supporting the conclusions of this article will be made available by the authors, without undue reservation.

## Ethics Statement

The studies involving human participants were reviewed and approved by SickKids Research Ethics Board. The patients/participants provided their written informed consent to participate in this study.

## Author Contributions

JS collected data, carried out the data analysis, drafted the initial manuscript, completed the analysis, interpretation of the data, and reviewed and revised the manuscript. JO, CW, SC, KK, and SM all provided substantial contribution to the conception, design of the study as well as the interpretation of the data, and critically reviewed and revised the manuscript. MA and AQ were involved in acquisition of the data and critically reviewed and revised the manuscript. RA conceptualized, designed the study, supervised the data analysis, interpretation of the data and critically reviewed, and revised all drafts of the manuscript. All authors drafted/revised the article for intellectual content, approved the final manuscript as submitted, and agree to be accountable for all aspects of the work.

## Funding

This work was supported by Norman Saunders Complex Care Grant.

## Conflict of Interest

The authors declare that the research was conducted in the absence of any commercial or financial relationships that could be construed as a potential conflict of interest.

## Publisher's Note

All claims expressed in this article are solely those of the authors and do not necessarily represent those of their affiliated organizations, or those of the publisher, the editors and the reviewers. Any product that may be evaluated in this article, or claim that may be made by its manufacturer, is not guaranteed or endorsed by the publisher.
