# Supplementary material for: Pediatric Chronic Tracheostomy Care: An Evaluation of an Innovative Competency-Based Education Program for Community Health Care Providers
Source: Front Pediatr. 2022 May 31;10:885405. doi: 10.3389/fped.2022.885405 (PMC9220937; doi:10.3389/fped.2022.885405)
Supplement: Supplementary file 1 [file Data_Sheet_1.pdf]

Module 1 – Paediatric Tracheostomy

Caring for Children Using Medical Technology

The Hospital for Sick Children/SickKids Learning Institute 2017

Program Description

This full day competency-based class will prepare the health care provider with the knowledge and skills to care for a child with a tracheostomy. Respiratory assessments, safety issues, oxygen therapy, care of the tracheostomy including suctioning, stoma care and tube changes, and managing urgent situations will be addressed. A pre-reading package is required to be completed prior to attending class in order to participate in discussions and learning activities. Participants will have the opportunity to practice skills and participate in a simulated home visit of a child with a tracheostomy.

Audience

Community health care providers including nurses, personal support workers and CYCs

Objectives

1. Review anatomy and physiology of the respiratory system
2. Identify the indications for receiving a tracheostomy
3. Demonstrate appropriate physical assessment of the respiratory system
4. Discuss the importance of maintaining a safe environment
5. Discuss oxygen therapy and humidification related to tracheostomies
6. Demonstrate skin and stoma care
7. Demonstrate proper technique when suctioning a tracheostomy tube
8. Demonstrate how to change a tracheostomy tube
9. Identify common urgent/emergent situations and management
   Provide safe, competent care for a patient with a tracheostomy in a simulation

Class Preparation Schedule

One month before class:

- ☐ Ensure adequate numbers of Paediatric Tracheostomy Care Resource handout

One week before class:

- ☐ Meet with simulation facilitators to review scenarios

Two days before:

- ☐ Print learner packages (PPT presentation, cases, agenda, handout)
- ☐ Print enough evaluation forms for each participant
- ☐ Print attendance sheet with names of registered participants
- ☐ Review powerpoint slides to ensure current content
- ☐ Create certificate of attendance documents

Class Agenda

0830-0845	Sign-in<br>Light refreshments will be served
0845-0900	Welcome and Introductions
0900-0945	Introduction to Paediatric Tracheostomy
0945-1130	Rotating Practice Stations<br>1. Oxygen Therapy, Humidification<br>2. Stoma Care, Tube Suctioning & Tracheostomy Tube Change<br><br>**Break will be provided after one rotation (1030-1045)**
1130-1215	Practice Stations<br>1. Troubleshooting Urgent/Emergent Situations
1215-1315	Lunch – on your own
1315-1600	Home Visit Simulations<br>• Four scenarios
1600-1630	Closing Remarks & Evaluation

Resources

Facilitation: This program is facilitated by an Interprofessional Education Specialist and Registered Respiratory Therapist. Simulation Education Specialists are required in the afternoon.

Physical space: Classroom style that can accommodate up to 16 people with ability to work in small groups

Pre-Reading materials: Paediatric Tracheostomy Care learning package

Technology: laptop/computer, LCD projector/screen, mannequins, portable suction, oximeter, simulation equipment

Implementation Plan

The program is to be delivered four times per year

Evaluation Methods

- Pre & post tests
- Course evaluation form

References

American Thoracic Society. (2000). Care of the child with a chronic tracheostomy. *American Journal Respiratory Critical Care Medicine, 161*, 297-308.

Bayliss, M., Hamp, C, Johnson, D., Leddy, R., Nonoyama, M. Robinson, C., & Turnbull, M. (Ed.) (2010). *Optimizing Respiratory Therapy Services A Continuum of Care from Hospital to Home.* College of Respiratory Therapists of Ontario, Canada. Retrieved from http://www.crto.on.ca/pdf/ProfPractice/HFO_Training_Manual.pdf

Bissell, C. (2013). *Aaron's Tracheostomy Page*. Retrieved from http://www.tracheostomy.com/

Boroughs, D.S. & Dougherty, J.M. (2015). Pediatric Tracheostomy Care: What home care nurses need to know. *American Nurse Today, 10* (3), 8-10.

College of Respiratory Therapists of Ontario. (2010). Home Ventilation and Tracheostomy Care: Teaching Manual for Paediatrics. In *Optimizing Respiratory Therapy Services: A Continuum of Care from Hospital to Home* (p.145-220). Retrieved from http://www.crto.on.ca/pdf/ProfPractice/HFO_Training_Manual.pdf

Deutsch, E.S. (2010). Tracheostomy: Pediatric Considerations. *Respiratory Care, 55* (8), 1082-1090.

Karapinar, B., Arslan, M. T. & Ozcan Coskan. (2008). Pediatric bedside tracheostomy in the pediatric intensive care unit: six-year experience. *The Turkish Journal of Pediatrics, 50*(4) 366-372

McClean, E.B. (2012). Tracheal Suctioning in children with chronic tracheostomies: A pilot study applying suction both while inserting and removing the catheter. *Journal of Pediatric Nursing 27*, 50-54.

Perry, S.E., Hockenberry, M.J., Lowdermilk,D. L., Wilson, D. Candian Editors: Sams, C. A. & Keenan-Lindsay, L. (2013). *Maternal Child Nursing Care in Canada.* Elsevier, Toronto, Canada.

Tabaee, A. et al. (2007). Practice patterns, safety, and rationale for tracheostomy tube changes: a survey of otolaryngology training programs. *The Larygoscope, 117*(4), 573-6.

Appendix

Additional documents:

Module 1 Paediatric Tracheostomy Learning Package

Tracheostomy Care Education Session Intro PowerPoint Presentation

Tracheostomy Care Practice Stations Facilitator Guide

Paediatric Tracheostomy Care Resource

Caring for a Child with a Tracheostomy Cases Facilitator Guide

Simulation Scenarios #1, 2 & 3AB and Observer Checklists

Certificate of Attendance

Course Evaluation Form

The Hospital for Sick Children/SickKids Learning Institute 2017

APPENDIX 2

Multiple Choice Tracheostomy Tube Care Knowledge Test

Dear Caregiver,
Thank you for taking the time to complete the questionnaire. SickKids is developing the Health Tech Junior course to best support healthcare providers in the community such as yourself. We need your help in this process to ensure that the course focuses on teaching the knowledge and skills that are needed most. Your responses will be kept anonymous. It will take approximately 5-10 minutes to complete this survey.
Please read and answer the questions below, choosing the answer you think is correct.

1. A Bivona pediatric tracheostomy tube can be reused how many times before it is thrown out?
   a) 0
   b) 1
   c) 3
   d) 4
   e) 5

2. Which of the following should be used to wash a Shiley (PVC) tracheostomy tube?
   a) Dish Soap and Boiled water
   b) Distilled Water
   c) Hydrogen Peroxide and sterile, distilled water
   d) Hot water that has been boiled and then cooled

3. You are caring for a 6 year old child with a tracheostomy at home. You go to insert the suction catheter and it doesn't pass. What would your next step be?
   a) Suction again with a smaller catheter
   b) Call 911
   c) Check the oxygen saturations
   d) Perform a tracheostomy change
   e) Remove the tracheostomy ties and check the stoma

4. You are caring for a 10 year old girl with Cerebral Palsy with a Pediatric Shiley 5.0 uncuffed tracheostomy tube. She starts to cough and her tracheostomy tube comes out of the stoma. You attempt to reinsert a Peds Shiley 5.0 tracheostomy tube but it won't go in. What do you do next?
   a) Check her oxygen saturations
   b) Call 911
   c) Try to re-insert the Peds Shiley 5.0 tracheostomy tube
   d) Cover the stoma with your thumb and start to manually bag the patient
   e) Try to re-insert a Peds Shiley 4.5 tracheostomy tube

5. How often should a Heated Moisture Exchanger (HME) be changed
   a) Daily if the child is using the HME 24 hours a day
   b) Every other day if the child is using the HME during the day only

c) If becomes soiled
d) If becomes plugged with mucous
e) All of the above

6. Which of the following are correct?
   a) When suctioning a 3.0 tracheostomy tube use a 8 French catheter
   b) When suctioning a 4.0 tracheostomy tube use a 10 French catheter
   c) When suctioning a 5.0 tracheostomy tube use a 12 French catheter
   d) When suctioning a 4.5 tracheostomy tube, use a 10 French catheter

7. What is the currently recommended frequency of cleaning the stoma?
   a) Once daily
   b) Twice daily
   c) Three times daily
   d) When there are visible secretions only
   e) After tracheostomy tube changes only

8. When cleaning the stoma, you should
   a) Wipe outward from the stoma
   b) Wipe inward from the stoma
   c) Apply Vaseline after stoma care is complete
   d) Wipe the stoma only if there are visible secretions

9. When should a manual resuscitation bag be used for a child with a tracheostomy tube?
   a) When the child has trouble breathing
   b) Before and after suctioning if needed
   c) If there is a problem with the ventilator
   d) If there is no power source available to operate the ventilator
   e) All of the above

10. You are completing the pressure test for the manual resuscitation bag. You squeeze the bag to empty it. You then cover the outlet of the bag with the palm of your hand. You release the bag while keeping the outlet covered with your hand. The bag does not fill up with air. What is the reason for this?
    a) There is a leak
    b) The manual resuscitation bag has expired
    c) The inlet valve may be sticking
    d) The PEEP valve may be malfunctioning

11. Which of the following are true regarding the use of a speaking valve?
    a) They can be used while a child is asleep
    b) They can be used when the tracheostomy tube has the cuff inflated
    c) They can improve the child's secretion management
    d) They cannot be used for infants

12. For a child with a Peds 4.0 Shiley tracheostomy tube, what should be in the emergency tracheostomy kit?

a) Peds Shiley 4.0, 3.5 ETT, gauze, normal saline, lubricant, scissors
b) Peds Shiley 4.0, Peds Shiley 3.0, gauze, normal saline, lubricant, scissors
c) Peds Shiley 4.0, Peds Shiley 3.5, gauze, normal saline, lubricant, scissors
d) None of the above

13. You are caring for a 2 year old girl with a Pediatric 3.0 Shiley tracheostomy tube. You notice that the secretions have gotten thicker. This may indicate which of the following?
    a) An early sign of infection
    b) The need for more frequent suctioning
    c) The need to increase humidification
    d) All of the above

14. Which of the following are indications that a child with a tracheostomy tube may need to be suctioned?
    a) Visible secretions or audible secretions
    b) Appears irritable, fussy
    c) Appears pale or blue
    d) a and c
    e) a, b and c

15. Which of the following are indications for a tracheostomy tube?
    a) Upper airway obstruction
    b) Pulmonary toileting
    c) Long-term Ventilation
    d) a and c
    e) a, b and c

16. You are caring for a 6 year old girl. She has just started to use a heated moisture exchanger (HME) throughout the entire day, and uses a tracheostomy mask at night. In the past week, she has had 6 mucous plugs in her tracheostomy tube during the day. What should be considered?
    a) Continue using the HME. The mucous plugs are a normal part of the acclimatization process
    b) Consider capping the tracheostomy tube during the day to prevent mucous plugs from forming
    c) Consider using the tracheostomy mask during the day or for parts of the day
    d) Consider using heated high flow humidity during the day
    e) C and D

17. Which of the following are correct regarding chest compressions during CPR?
    a) For infants, compression depth is 1.5 inches (4cm)
    b) For children, compression depth is 2 inches (5cm)
    c) For teens, compression depth is 2-2.4 inches (5-6cm)
    d) All of the above
    e) None of the above

18. Which of the following are true regarding speaking valves?
    a) Speaking valves should be cleaned with a solution of vinegar and water
    b) All speaking valves should make a vibrating sound
    c) The valves should be checked to make sure they are not sticking

d) If your patient is using a cuffed tracheostomy tube, the speaking valve should be used with the cuff up
e) Speaking valves can be used during daytime naps

19. When caring for a child with a tracheostomy tube, which of the following is true
    a) 24/ 7 eyes on care
    b) suction machine should always be on hand
    c) an HME is recommended during bathing
    d) CPR skills of the caregiver should be recertified every 18 months
    e) a,b,c
    f) a,b,c,d

20. You are caring for a 5 year old boy with a tracheostomy tube. At the start of your shift while you are doing your initial assessment, you notice that the child is unresponsiveness and there is no definite pulse. You are alone and do not have a cell phone. What are your next steps?
    a) call 911
    b) start doing 2 minutes of CPR at a ratio of 30 chest compressions: 2 breaths before calling for help
    c) start doing 2 minutes of CPR at a ratio of 15 chest compressions: 2 breaths before calling for help
    d) none of the above

APPENDIX 3

Self-Efficacy Questionnaire

Please rate how certain you are that you can provide the following aspects of tracheostomy care for a **PEDIATRIC** client (infant or child) **as of now**.

Rate your degree of confidence by recording a number from 0 to 100 using the scale given below:

0	10	20	30	40	50	60	70	80	90	100
Cannot do at all					Can do moderately					Highly certain can do

**Knowledge and Skills**	**Confidence (1 -100)**
**Assessment**	
Conduct a respiratory assessment on a pediatric client	____________
Recognize respiratory distress in a pediatric client	____________
**Safety**	____________
Assess securement of a tracheostomy tube	____________
Perform equipment checks (oximeter, suction machine, oxygen)	____________
Ensure emergency tracheostomy kit is complete	____________
Check the manual resuscitation bag	____________
**Tracheostomy Care**	____________
Provide stoma care	____________
Demonstrate changing a tracheostomy tube	____________
Demonstrate suctioning of a tracheostomy tube	____________
Administer medication via a tracheostomy tube	____________
**Troubleshooting Emergent/Urgent Situations**	____________
Provide oxygen therapy to a deteriorating pediatric client with a tracheostomy tube	____________
Manage a blocked tracheostomy tube	____________
Manage an accidental decannulation of a tracheostomy tube	____________
Provide manual ventilation via a tracheostomy tube	____________
Provide bag-mask ventilation to a pediatric client	____________
Perform CPR on a pediatric client according to the current standards of the Heart and Stroke Foundation of Canada	____________
